# COVID-19 Booster Vaccine Intention by Health Care Workers in Jakarta, Indonesia: Using the Extended Model of Health Behavior Theories

**DOI:** 10.3390/tropicalmed7100323

**Published:** 2022-10-21

**Authors:** Suzy Maria, Dicky C. Pelupessy, Sukamto Koesnoe, Evy Yunihastuti, Dwi Oktavia T. L. Handayani, Tommy Hariman Siddiq, Astri Mulyantini, Ahmad Rhyza Vertando Halim, Endang Sri Wahyuningsih, Alvina Widhani, Ghina Shabrina Awanis, Maulana Girsang Muda, Teguh Harjono Karjadi, Anshari Saifuddin Hasibuan, Iris Rengganis, Samsuridjal Djauzi

**Affiliations:** 1 Allergy and Clinical Immunology Division, Department of Internal Medicine, Faculty of Medicine, Universitas Indonesia/Cipto Mangunkusumo Hospital, Jakarta 10430, Indonesia; 2 Faculty of Psychology, Universitas Indonesia, Depok 16424, Indonesia; 3 DKI Jakarta Provincial Health Office, Jakarta 10160, Indonesia; 4 Faculty of Psychology and Education, Universitas Al-Azhar Indonesia, Jakarta 12110, Indonesia; 5 Cipto Mangunkusumo Hospital, Jakarta 10430, Indonesia

**Keywords:** COVID-19, booster vaccine, theory of planned behavior, health belief model, intention, vaccine hesitancy

## Abstract

In Indonesia, COVID-19 vaccination hesitancy persists among health care workers (HCWs). Understanding the motives and challenges impacting HCWs’ acceptance of the booster vaccination is critical. Efforts are still needed to overcome apprehension about taking a booster dosage. This study aims to analyze the vaccine acceptance among HCWs in Jakarta using an extended, modified model of health behavior theories, namely The Health Belief Model (HBM) and The Theory of Planned Behavior (TPB). A cross-sectional survey from November 2021 to January 2022 was distributed to health care workers in Jakarta. Bivariate analysis followed by multivariate regression was used to assess factors associated with the vaccine intention and collected 1684 responses. The results have shown that the final model combining the constructs and demographic characteristics could explain 50% of the variance of intention to receive a COVID-19 booster vaccination. Moreover, anticipated regret had the most significant standardized coefficient among the constructs (β = 0.381, *p* < 0.001). Other significant predictors in the model were attitude (β = 0.243, *p* < 0.001), perceived benefits (β = 0.103, *p* < 0.001), subjective norms (β = 0.08, *p* = 0.005), and perceived susceptibility (β = 0.051, *p* = 0.016). The findings can be used to strategize interventions to increase vaccine uptake.

## 1. Introduction

Coronavirus disease 2019 (COVID-19) has brought a global catastrophe in the health and socioeconomic fields. Indonesia bears a heavy burden, where infection and mortality are some of the highest in Southeast Asia. One of the greatest hopes for stopping the pandemic is a vaccine. Efforts to develop a vaccine against SARS-CoV-2 have been a resounding success, producing multiple modalities in a brief period [1,2,3,4].

According to the WHO Strategic Advisory Group of Experts on Immunization (SAGE) Roadmap, priority populations for vaccine recipients are health workers with a high risk of infection and transmission of SARS-CoV-2 in the community and those who are susceptible to severe infection, even leading to death, when infected with SARS-CoV-2 [4].

In July 2021, the Indonesian Ministry of Health started administering a COVID-19 booster vaccine for all health care workers, considering the conditions cannot provide adequate immunity against the mutated SARS-CoV-2 variant [5]. However, four months later, during this study in November 2021, only 78% of health care workers in Indonesia were vaccinated with the COVID-19 booster vaccine [6].

The most critical public health approach for controlling the pandemic is to achieve very high vaccination coverage for primary and booster doses. The nationwide research of HCWs during the initial distribution revealed that hesitation persists among HCWs [7]. Understanding the motives and challenges impacting the acceptance of the booster vaccination is critical. Efforts are still needed to overcome apprehension about taking a booster dosage [8]. Published studies on the HCW population in other countries have mentioned a few reasons for booster vaccine hesitancy: concerns about vaccine safety, distrust of the government, and fear of adverse reactions [8,9].

Due to the various characteristics of the condition, addressing vaccine hesitancy in a specific demographic may be important [10,11]. Understanding the factors on Indonesian HCWs’ intentions to get the COVID-19 booster vaccine is crucial because these factors can differ significantly by location, culture, and socioeconomic level.

Conceptual behavior frameworks containing primary constructs are essential for understanding the elements influencing decision-making by determining what motivates and prevents people from engaging in health-related action. The Health Belief Model (HBM) was established in the 1950s to explain the widespread failure of illness prevention and detection initiatives [12,13]. The premise of HBM is that people are more likely to engage in a health behavior when at risk for a disease, the condition may have potentially serious consequences, and the course of action available may be beneficial in reducing their susceptibility. The HBM clearly states that health beliefs collectively influence actions, but no specific combinations, weights, or connections between variables are specified. As a result, studies have begun integrating HBM elements with constructs from other theories [12].

The Theory of Planned Behavior (TPB) is an extension of the Theory of Reasoned Action (TRA). The primary concept of the TRA/TPB is that behavioral intention is the most significant predictor of behavior. Individuals’ attitudes toward completing the activity and subjective norms connected with behavior are direct drivers of their behavioral intentions. TPB incorporates perceived control over a specific behavior, accounting for scenarios where one may not have total volitional control over the action [12].

The integrated model of health behavior combines elements from popular theories and has been utilized in the vaccination acceptance research. Le An et al. discovered that a model combining demographics and HBM and TPB factors predicted the desire to obtain the vaccination, accounting for 39% of the variation [14]. Shmueli used HBM and TPB on the general public in Israel and found that the model could explain 78% of the variance in the intention to receive a COVID-19 vaccine, which was considerably higher than using each of the two behavior models separately [13].

This study aims to analyze booster vaccine acceptance using the integrated model of health behavior theories. The findings can be used as a basis for health policies regarding the COVID-19 booster vaccine for the broader HCW population.

## 2. Materials and Methods

This cross-sectional study was conducted from November 2021 to January 2022. The subjects were all health care workers in DKI Jakarta Province who received the primary vaccine.

Data were collected using an independent website in Bahasa Indonesia. The online consent form included a disclaimer, where participation was voluntary, and no penalties were involved for refusing to participate. This study was conducted per the Declaration of Helsinki and was approved by the Faculty of Medicine Ethics Committee, Universitas Indonesia.

Cochran’s sample size formula for categorical data for an alpha level a priori at 0.05 was used to estimate sample size. The sample size needed for this study is found to be 1067.

### 2.1. Survey Measures

The authors developed the survey used in this study. Elicitation interviews were done at first as part of the survey making. Individual qualitative interviews with ten HCWs were conducted in their native languages and were structured around the components of HBM and TPB. Participants were asked to consider receiving a COVID-19 booster vaccine and report their sentiments and views regarding the vaccine’s outcomes, sources of normative influence, and obstacles and facilitators to vaccination.

The content analysis of transcribed interviews revealed lists of participants’ sentiments, behavioral outcomes, sources of normative influence towards vaccination, and obstacles and facilitators to vaccination. Additionally, we measured the internal consistency of each item for the construct validation process. We chose items with internal consistency higher than 0.3.

Following a content analysis, the items of the corresponding constructs were finalized. The final survey questionnaire included sections on socio-demographic characteristics.

The questionnaire consisted of three main sections. The first one included demographic information such as age, gender, marital status, the highest level of education, history of COVID-19 infection, history of comorbidity, and contact with patients at work. The intention was measured using a single question, and *I intend to get a booster COVID-19 vaccination* was rated from *highly disagree* (1) to *agree* (5). 

The second section included questions based on extended HBM theory, encompassing attitude, subjective norms, self-efficacy, perceived control, and anticipated regret.

*Attitude.* To understand the participants’ attitudes towards the vaccination, participants were asked to answer a semantic differential scale ranging from 1 to 5 (“For me, getting a booster COVID-19 vaccination would be … *Very bad, good, wrong, wise, harmful–highly beneficial, disappointing–satisfying, and negative–very positive*”). Scores were summed to constitute a measure of attitude (α = 0.952).

*Subjective norms* were assessed by four items ranked from *highly disagree* (1) to *highly agree* (5). The items used were ‘My immediate family will *(highly disagree–highly agree)* on my decision to conduct the COVID-19 booster vaccination’, ‘My close friends will *(highly disagree–highly agree)* on my decision to carry out the COVID-19 booster vaccination’, ‘My teammates at work will *(highly disagree–highly agree)* on my decision to carry out the COVID-19 booster vaccination’, and ‘The important people in my life will *(highly disagree–highly agree)* on my decision to carry out the COVID-19 booster vaccination’. The scores were added together to form a measure of subjective norms (α = 0.946).

*Self-efficacy.* Three items measured self-efficacy: ‘For me, carrying out a booster vaccination will be *(very easy–very difficult)*’, ‘I am confident that I can conduct the vaccination *(highly disagree–highly agree)*’, and ‘when I want, I can easily get a vaccination *(highly disagree–highly agree)*’ (α = 0.724).

*Perceived control.* Four questions were asked to constitute a measure of perceived control: ‘The decision to conduct the vaccination is beyond my control *(highly disagree–highly agree)*’, ‘How many things are beyond your control that would prevent getting the vaccination *(very few–too many)*’, ‘It is all up to me to obtain a booster vaccination or not *(highly disagree–highly agree)*’, and ‘What is your role in determining the COVID-19 booster vaccination? *(very huge–very little)*’ (α = 0.667).

*Anticipated regret.* The additional construct to the TPB theory, anticipated regret, was measured by the question, ‘since I cannot obtain the vaccination at this time, I want to get it in the future, ranked from *highly disagree* (1) to *agree* (5).

The last section, the domain of HBM theory, consisted of 19 items categorized into four constructs, scored on a five-point Likert scale from *‘highly disagree’ (score: 1) to ‘agree’ (score: 5)*. The constructs included are:

*Perceived susceptibility.* This construct was measured with four items, namely *The possibility of me being infected with COVID-19 is quite high, I’m worried that my family members will catch COVID-19, The possibility of my co-workers being infected with COVID-19 is quite high,* and *I am worried that I will be infected with COVID-19 in the future even though I have been vaccinated twice*. Cronbach’s alpha = 0.878.

*Perceived severity.* The summation of five items measured this construct, with α = 0.764 are *I am afraid of being infected with COVID-19 after the vaccine, I am worried that the first and second vaccines received are not effective against the new variants, I’m worried about the side effects of this booster vaccine, I’m worried about complications post COVID-19 infection affecting my daily activities,* and *I can’t be vaccinated because of the disease I have.*

*Perceived benefit.* Items used to represent this construct are *The COVID-19 booster vaccination makes me feel safe at work, vaccination reduces my chances of contracting COVID-19, Booster vaccinations can reduce my chances of getting infected with the new variant, vaccination can reduce complications caused by COVID-19,* and *The booster will keep me working and earning* (α = 0.783).

*Perceived barrier.* The sum of the scores of these five items was used to illustrate perceived barriers, namely, *I’m worried about experiencing stress because of being infected, The COVID-19 booster vaccination saves me from stress due to self-isolation, I cannot be vaccinated because I just recovered, I cannot be vaccinated because my data was not recorded in the data system,* and *I cannot be vaccinated because I’m pregnant and breastfeeding* (α = 0.862).

### 2.2. Data Analysis

Data were analyzed using SPSS^®^ Statistics 25 software (IBM Corp., Armonk, NY, USA). Descriptive statistics were reported using frequency and percentages for categorical data and mean and standard deviation for numeric data. The Chi-square or Fisher’s exact test were used in the bivariate analysis to evaluate the association between the primary outcome variable (intent to get COVID-19 booster vaccine) and independent variables. A two-step hierarchical regression analysis was conducted to test for predictors of vaccination intentions. A *p*-value < 0.05 was considered statistically significant.

## 3. Results

### 3.1. Background Characteristics

In total, 1684 responses were collected from all the cities in DKI Jakarta. About 89.3% of respondents intended to obtain a booster vaccination (scores above 3 on a scale from 1 to 5). Only 1.7% of participants did not get vaccinated (scores below 3), and 9% chose ‘neutral’ in response to the question ‘I intend to get a booster COVID-19 vaccination’. Table 1 summarizes the respondents’ demographic, socioeconomic, and other background information.

A probable link between participants’ desire to vaccinate and four characteristics were discovered using a bivariate statistical analysis (*p* < 0.05; Table 1). With higher education and income, the intention to be vaccinated increased significantly. Intention to be vaccinated is also higher in respondents with a history of infection and comorbidity. Table 1 shows the specifics of the above findings.

### 3.2. Regression Analysis

The final model (Table 2) combining the constructs and demographic characteristics shows that 50% of the variance of intention to receive the vaccination was explained by the combination of the constructs and demographic variables. In the model, anticipated regret had the most significant standardized coefficient among the constructs. Furthermore, a one-unit increase in behavior belief increased the intention of getting the COVID-19 booster vaccine by 0.381 units (β = 0.381, *p* < 0.001). Other significant predictors were attitude (β = 0.243, *p* < 0.001), perceived benefits (β = 0.103, *p* < 0.001), subjective norms (β = 0.08, *p* = 0.005), and perceived susceptibility (β = 0.051, *p* = 0.016).

## 4. Discussion

This study analyzes the factors influencing the intention of health care workers to get a booster vaccination. The results can aid policymaking to improve the booster vaccination rate in the capital city of Indonesia, which is still being hit by the COVID-19 pandemic. Furthermore, this is the first study analyzing factors related to the intention of getting the COVID-19 booster vaccine in Indonesia.

The study found high booster vaccination acceptance among HCWs in Jakarta, with 89.3% agreeing to take the vaccine. This number is higher than a previous study among HCWs analyzing the intention to get the primary shots, which reported that only 61.3% of participants were willing to get the vaccine [7]. After the previous study, more evidence of the COVID-19 vaccine being safe and effective was released, and hesitancy has been known to be a matter of time and context [15].

The number of HCWs agreeing to the vaccine is also considerably higher than the number reported in other countries. In a study by Vellappally et al., only 64% of HCWs were willing to receive the vaccination in Saudi Arabia, while the same study reported 84% in India [9]. This is also higher than 56.3% of respondents accepting the vaccine in Jakarta and Bali, as reported by Wirawan et al. [16].

The anticipated regret is the most significant predictor of HCW’s intention to receive the vaccine in Jakarta. Anticipated regret aims to analyze the inaction regret of not getting the vaccine, and the finding is also confirmatory with previous studies [17,18]. In a meta-analysis by Brewer et al., anticipated inaction regret is more strongly felt than action regret and has more reliable associations with behavioral intentions [19]. Health behavior intervention regarding the vaccine should emphasize the consequences of inaction and elicit self-regret. Considering the high primary vaccination rate, the anticipated regret is driven by the feeling of not following societal pressure from peers and the government.

In this study, attitude towards a booster vaccine is also a significant predictor of the intention to vaccinate. The finding is in line with previous studies focusing on the attitude toward the vaccine [9,20]. Promoting vaccination with clear information and combating hoaxes and misinformation might change the previously negative attitude towards a booster vaccine.

Intervention toward hesitance should highlight the benefits of getting a booster vaccine. In this study, HCW considers perceived benefits as a significant driver towards getting a booster vaccine. Policymakers may consider employing messages emphasizing the usefulness and advantages of receiving the booster, consistent with intervention research that employs behavior modification strategies to change attitudes [21].

Items related to subjective norms, a construct that signifies the relevance of other people’s views, are also significant. The decision to vaccinate was influenced by the opinions of co-workers and close friends regarding the vaccine [22,23,24]. Health care workers are susceptible to COVID-19 even after primary vaccination. This could also be why HCWs have a high sense of perceived susceptibility, affecting their intention.

The socioeconomic characteristics are not considered significant in the final regression model. In the bivariate analysis, HCWs with higher education levels are more likely to be vaccinated. However, these characteristics are insignificant when combined with the behavioral constructs, as reported by Dziedic et al. [25]. The booster vaccination is a personal choice driven by intrinsic factors and not influenced by socioeconomic class. Highlighting this finding could be useful for policymakers, as mass media and large-scale intervention should promote vaccine intake.

This study is the first to report COVID-19 booster vaccine acceptance involving HCW with a sizable sample size. Furthermore, it is one of the first in Indonesia to report an intention to get COVID-19 booster vaccination and acceptance. It is the first to combine HBM and TPB constructs to analyze vaccination intentions.

Concerning the limitations, there has been a sampling bias, since the study was conducted through an internet questionnaire. Some people could not participate because they had no smartphone or internet connection. Second, the validity of the responses was not easily ensured because the study did not survey a person. Moreover, this study is cross-sectional, and the booster vaccination varies in response to the pandemic condition. Therefore, this study may not wholly reflect the most recent intention of the Jakarta HCW population to receive the booster vaccination.

## 5. Conclusions

This study has shown the usefulness of the model of health behavior theories to explain predictors of intention to obtain COVID-19 booster vaccination among the HCW population in Jakarta, Indonesia. As this study aims to confirm the robustness of a model derived from health behavior theories to measure intention, future research can delve into how this model relates to actual behavior. Stakeholders, in this case, the DKI Jakarta Health Provincial Office, can also use the results of this study in terms of policymaking. This study highlighted relevant issues and barriers to COVID-19 booster vaccination that can be used as a reference to increase booster vaccine uptake.

## Figures and Tables

**Table 1:** Number and percent distribution of participants’ levels of agreement to the statement reflecting intentions for booster shots, Jakarta, 2021–2022.

Characteristics	Answer to ‘I Intend to Get A Booster COVID-19 Vaccination’					Total	*p*-Value
	Highly Disagree	Disagree	Neutral	Agree	Highly Agree		
Age group							0.431
18–59	16 (1%)	12 (0.7%)	151 (9.2%)	355 (21.6%)	1110 (67.5%)	1644 (97.6%)	
≥60	0 (0%)	0 (0%)	1 (2.5%)	7 (17.5%)	32 (80%)	40 (2.4%)	
Sex							0.848
Male	5 (1.1%)	3 (0.7%)	43 (9.9%)	87 (20%)	298 (68.3%)	436 (25.9%)	
Female	11 (0.9%)	9 (0.7%)	109 (8.7%)	275 (22%)	844 (67.6%)	1248 (74.1%)	
Marriage status							0.215
Married	15 (1.3%)	10 (0.8%)	103 (8.6%)	254 (21.3%)	812 (68%)	1194 (70.9%)	
Others (single, divorced)	1 (0.2%)	2 (0.4%)	49 (10%)	108 (22%)	330 (67.3%)	490 (29.1%)	
Education							<0.001 *
High school or lower	8 (1.2%)	7 (1%)	99 (14.8%)	188 (28%)	369 (55%)	671 (39.8%)	
Diploma or higher	8 (0.8%)	5 (0.5%)	53 (5.2%)	174 (17.2%)	773 (76.3%)	1013 (60.2%)	
Income							<0.001 *
Provincial minimum wage or lower (<=296 USD)	9 (1.5%)	6 (1%)	79 (13.1%)	150 (24.8%)	360 (59.6%)	604 (35.9%)	
1–3x more than the provincial minimum wage (297–888 USD)	6 (0.8%)	5 (0.6%)	66 (8.4%)	172 (22%)	534 (68.2%)	783 (49.5%)	
>3x more than provincial minimum wage (>888 USD)	1 (0.3%)	1 (0.3%)	7 (2.4%)	40 (13.5%)	248 (83.5%)	297 (17.6%)	
History of COVID-19							0.011 *
No	10 (1.1%)	6 (0.7%)	66 (7.3%)	196 (21.7%)	624 (69.2%)	902 (53.6%)	
Yes, before vaccination	3 (0.8%)	4 (1%)	37 (9.5%)	75 (19.2%)	271 (69.5%)	390 (23.2%)	
Yes, after the first vaccination	1 (1.4%)	2 (2.7%)	14 (19.2%)	12 (16.4%)	44 (60.3%)	73 (4.3%)	
Yes, after the second vaccination	2 (0.6%)	0 (0%)	35 (11%)	79 (24.8%)	203 (63.6%)	319 (18.9%)	
Number of COVID-19 Infection History							0.619
0	14 (0.9%)	12 (0.8%)	137 (8.7%)	343 (21.7%)	1075 (68%)	1581 (93.9%)	
1	2 (2.3%)	0 (0%)	12 (14%)	15 (17.4%)	57 (66.3%)	86 (5.1%)	
2	0 (0%)	0 (0%)	2 (16.7%)	4 (33.3%)	6 (50%)	12 (0.7%)	
3 times or more	0 (0%)	0 (0%)	1 (20%)	0 (0%)	4 (80%)	5 (0.3%)	
Any comorbidity							0.001 *
Yes	2 (0.6%)	2 (0.6%)	12 (3.3%)	78 (21.7%)	265 (73.8%)	359 (21.3%)	
No	14 (1.1%)	10 (0.8%)	140 (10.6%)	282 (21.3%)	877 (66.3%)	1325 (78.7%)	
Contact COVID-19 patients at work							0.169
Yes	10 (1%)	6 (0.6%)	80 (7.8%)	218 (21.2%)	715 (69.5%)	1029 (61.1%)	
No	6 (0.9%)	6 (0.9%)	72 (11%)	144 (22%)	427 (65.2%)	655 (38.9%)	

* *p*-value < 0.05, calculated using Chi-square test or Fisher’s exact.

**Table 2:** Results from regression analysis combining the constructs and significant demographic characteristics (education, income, history of COVID-19, and comorbidity).

Variables	*B*	*SE B*	β	*R^2^*
Model 1				0.496
Anticipated regret	0.408 *	0.023	0.382	
Attitude	0.059 *	0.007	0.251	
Perceived benefits	0.021 *	0.004	0.103	
Subjective norms	0.025 *	0.008	0.085	
Perceived susceptibility	0.011 *	0.004	0.052	
Perceived severity	−0.001	0.004	−0.006	
Perceived barrier	0.001	0.004	0.006	
Perceived control	−0.006	0.007	−0.014	
Self-efficacy	0.006	0.009	0.013	
Model 2				0.5
Anticipated regret	0.406 *	0.023	0.381	
Attitude	0.058 *	0.007	0.243	
Perceived benefits	0.021 *	0.004	0.103	
Subjective norms	0.024 *	0.008	0.080	
Perceived susceptibility	0.01 *	0.004	0.051	
Perceived severity	−0.001	0.004	−0.006	
Perceived barrier	0.002	0.004	0.01	
Perceived control	−0.005	0.007	−0.013	
Self-efficacy	0.007	0.009	0.017	
Education (high school or less)	−0.051	0.03	−0.033	
Income (minimum wage or lower)	0.01	0.043	0.006	
Income (>1–3x more than minimum wage)	−0.003	0.039	−0.002	
History of COVID-19 (yes, before vaccination)	0.033	0.033	0.018	
History of COVID-19 (yes, after the first vaccination)	−0.116	0.067	−0.031	
History of COVID-19 (yes, after second vaccination)	−0.055	0.036	−0.028	
Any comorbidity (yes)	0.047	0.033	0.025	

* *p*-value < 0.05.

## Data Availability

The data used are available from the corresponding author upon request.
